# Steroidogenic Factor 1 (SF1) Immunohistochemical Stain Is Sensitive and Specific for the Cytopathologic Identification of Intrapancreatic Ectopic Splenic Tissue

**DOI:** 10.1002/dc.70093

**Published:** 2026-02-02

**Authors:** Adeyinka Akinsanya, Jessica L. Muldoon, Mohamed Mustafa, Bin Yang, Hector Mesa, Omer Saeed

**Affiliations:** ^1^ Department of Pathology Indiana University School of Medicine Indianapolis Indiana USA; ^2^ Diagnostic Institute, Cleveland Clinic Lerner College of Medicine Cleveland Ohio USA; ^3^ University of Maryland School of Medicine Baltimore Maryland USA

**Keywords:** accessory spleen, fine needle aspiration, immunohistochemistry, pancreas, SF1, spleen, steroidogenic factor

## Abstract

**Background:**

Accessory spleens result from failure of splenic fusion during development and are occasionally located in the pancreatic tail, radiographically appearing as solid or solid/cystic lesions. Fine needle aspiration (FNA) easily identifies accessory spleens in most samples based on architecture and using CD8 to highlight splenic sinusoidal cells. However, in small samples, the architecture is not readily appreciated, and cross‐reactivity with CD8+ T‐cells can create diagnostic challenges. The purpose of the study is to evaluate the utility of steroidogenic factor 1 (SF1) immunohistochemical (IHC) staining in identifying accessory spleens.

**Methods:**

A retrospective search of FNA samples from 2010 to 2023 was performed to collect cases signed out as or consistent with intrapancreatic accessory spleen or splenule. Comparison tissue was collected from the regional lymph nodes. Tissues with no or hypocellular cell blocks were excluded from the study. IHC stains CD8 and SF1 (EP434) Rabbit Monoclonal Primary Antibody were performed on the cell blocks.

**Results:**

The intrapancreatic accessory spleens (13 cases) were universally positive for both cytoplasmic CD8 and nuclear SF1 in a sinusoidal pattern. The regional lymph nodes (15 cases) showed positivity for CD8 in background lymphocytes (scattered) and were universally negative for SF1.

**Conclusions:**

While CD8 and SF1 are comparable in their sensitivity, SF1 has benefits in cytologic samples. As a nuclear stain, SF1 is more easily interpreted and better suited for cell transfers. SF1 also eliminates the background CD8+ T cell staining that can be present in lymphoid tissue. Overall, utilization of SF1 can be beneficial in the cytologic workup of an intrapancreatic accessory spleen.

## 1 Introduction

Steroidogenic factor 1 (SF1) is a nuclear transcription factor critical to the regulation of steroidogenesis and reproductive development [1]. It is predominantly expressed in steroidogenic tissues, including the adrenal cortex and gonads, where it plays a key role in the biosynthesis of steroid hormones. SF1 is also expressed in splenic endothelial cells, where it plays a crucial role in the development of the splenic venous sinuses and pulp veins. This is a less recognized but diagnostically significant feature [1, 2]. Mutations in SF1 can lead to asplenia, a condition characterized by the absence of a spleen [3]. Ectopic splenic tissue, particularly accessory spleens located within or adjacent to the pancreatic tail, is often incidentally discovered on imaging and can mimic primary pancreatic neoplasms or enlarged lymph nodes radiologically [4]. As a result, sampling and diagnosing them adequately is of paramount importance to guide clinical management. Endoscopic ultrasound‐guided fine‐needle aspiration (EUS‐FNA) is the preferred diagnostic modality for evaluating pancreatic tail lesions, owing to its ability to sample deep‐seated areas with minimal risk [5]. However, the limited cellularity and distorted architecture inherent to some FNA specimens can pose diagnostic challenges, highlighting the need for specific and reliable immunohistochemical (IHC) markers. In the normal spleen, a specialized subset of endothelial cells known as littoral cells (LCs) exhibits distinctive features, including the expression of the scavenger receptor CD8α/α, which can be reliably identified using the CD8 immunostain [6]. This stain is commonly used for the recognition of sinusoids in FNA cytology. The use of SF1 IHC stain for the diagnosis of ectopic spleen has not been reported. In this study, we present a series of 13 cases of intrapancreatic accessory spleen diagnosed by FNA, in which SF1 IHC staining was performed alongside a panel of other IHC markers. To our knowledge, this represents the largest and only reported series to date evaluating the diagnostic utility of SF1 in the identification of ectopic splenic tissue in cytology specimens.

## 2 Materials and Methods

This retrospective study was conducted with the approval of the institutional review board at Indiana University. A search of the pathology database was performed to identify all cytology cases diagnosed as consistent with intrapancreatic accessory spleen or splenule between January 2010 and December 2023. For comparison, cytology specimens from benign lymph nodes over the same period were also collected. Cases with absent or inadequate hypocellular cell blocks were excluded from the study. Hypocellular cell blocks were defined by either an absence of cells (acellular) or the presence of only scattered inflammatory cells and/or contaminating mucosa. The relevant clinical history was extracted from the electronic medical record for all included cases. Cytologic material available for review included Papanicolaou‐stained smears; air‐dried, Diff‐Quik–stained smears; hematoxylin and eosin (H&E)–stained cell block sections; and any previously performed IHC stains.

IHC stains for SF1 (clone EP434, Rabbit Monoclonal Primary Antibody) and CD8 (clone C8/144B, CD8 Agilent antibody) were performed on cell blocks of all identified cases, as well as the comparison lymph node samples. SF1 was considered positive when there was strong nuclear staining, while CD8 positivity was determined by cytoplasmic staining in splenic endothelial cells lining sinusoidal spaces. Background staining of T‐lymphocytes was disregarded.

## 3 Results

**FIGURE 1:**
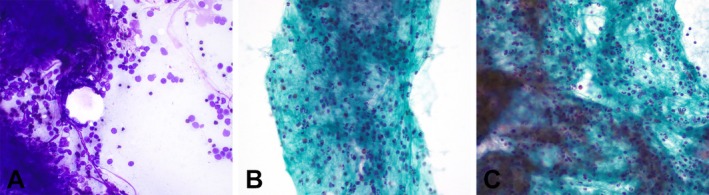
Fine needle aspiration of an intrapancreatic accessory spleen at 400× magnification. (A) Loosely cohesive cluster of mixed inflammatory infiltrate predominantly made up of polymorphous lymphocytes (Diff‐Quik stain). (B, C) Characteristic eosinophils, admixed with other inflammatory cells (Papanicolaou stain). [Color figure can be viewed at wileyonlinelibrary.com]

This study included 13 patients with intrapancreatic ectopic spleens diagnosed through FNA. The clinical, radiologic, and cytologic characteristics of these cases are summarized in Table 1. The median age of the patients was 63 years (range: 35–79 years), with 6 men (46%) and 7 women (54%) included in the study. The most common site for an ectopic spleen was the pancreatic tail, in 12 cases (92%). The remaining accessory spleen was located within the left upper quadrant soft tissue (8%). The median size of the lesions was 15 mm (range: 7–45 mm). Cell blocks were available in all 13 cases. Prior IHC stains were performed in 4 cases (31%), and flow cytometry in one case. Cytologic evaluation on the Diff‐Quik and Papanicolaou‐stained smears showed predominance of small, mature‐appearing lymphocytes centered around delicate thin vessels as well as scattered eosinophils (Figure 1). On cell block sections, the lymphocytes were often interspersed with open spaces, morphologically consistent with splenic sinusoids. For comparison, 15 cases from lymph nodes obtained over a similar time frame were also reviewed to assess staining patterns in the vasculature.

**TABLE 1:** Clinical and pathologic characteristics of accessory spleen cases, *N* = 13.

Characteristic	No. of cases (%)
Patient demographics	
Age, median, range (years)	63, (35–79)
Sex	
Male	6 (46)
Female	7 (54)
Site of accessory spleen	
Pancreatic tail	12 (92)
Left upper quadrant soft tissue	1 (8)
Size of lesion, median, range (mm)	15, (7–45)
Cases with cell block	13 (100)

**FIGURE 2:**
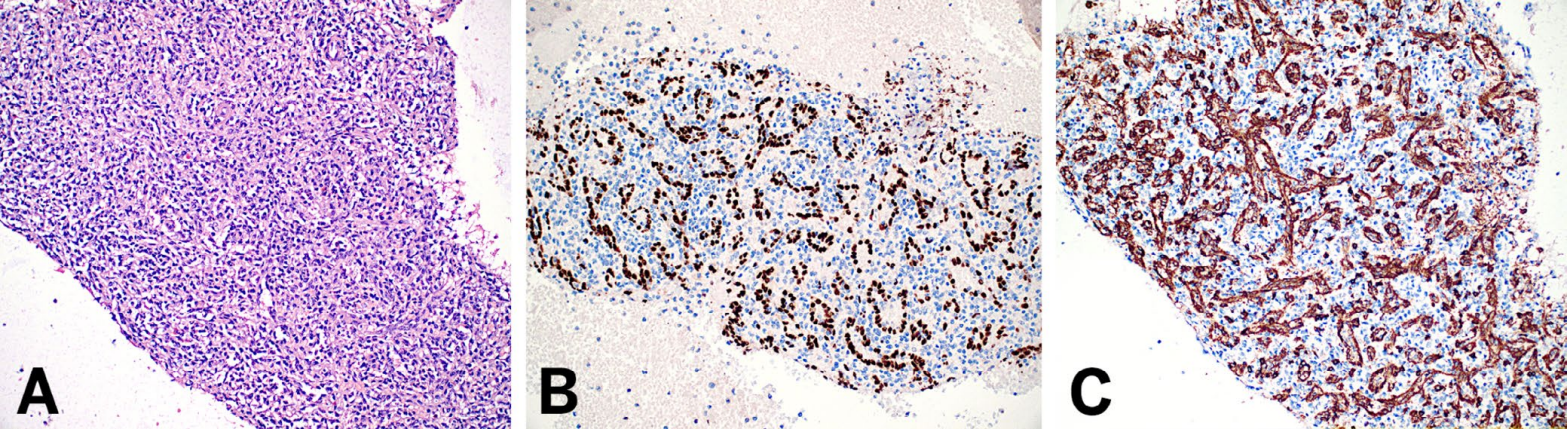
Immunohistochemical features of ectopic spleen at 200× magnification. (A) Hematoxylin and eosin (H&E)‐stained cell block showing splenic tissue. (B, C) SF1 and CD8 immunostains highlight splenic sinusoidal cells respectively. [Color figure can be viewed at wileyonlinelibrary.com]

**FIGURE 3:**
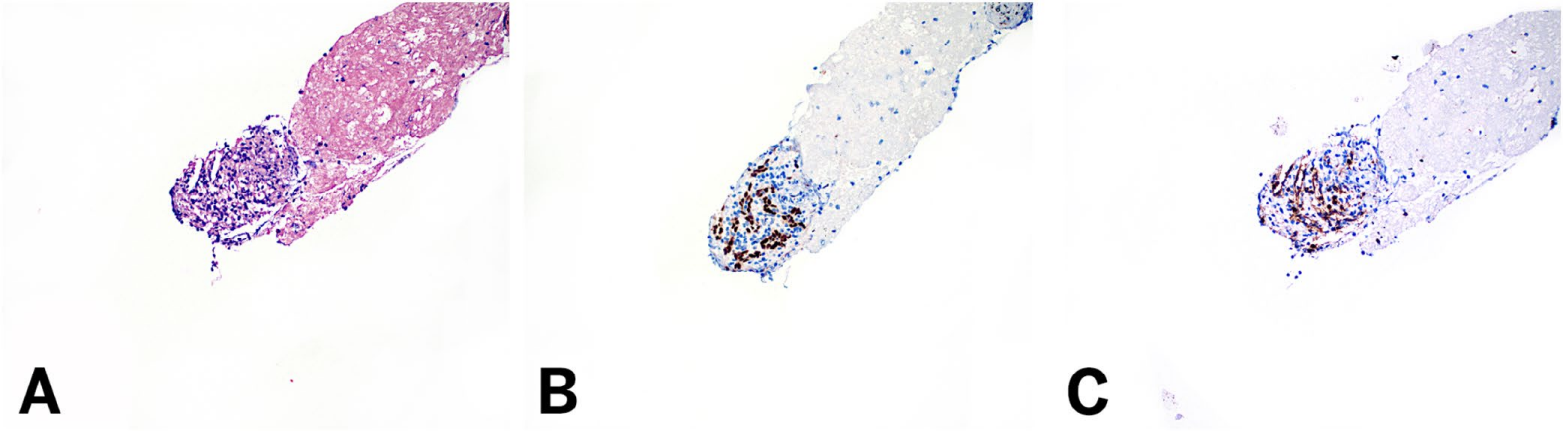
Limited cellularity ectopic spleen sample at 100× magnification. (A) Cell block showing scant lymphoid cells. (B) Splenic endothelial cells highlighted by SF1 stain. (C) CD8 shows patchy cytoplasmic staining in the splenic endothelium. [Color figure can be viewed at wileyonlinelibrary.com]

**FIGURE 4:**
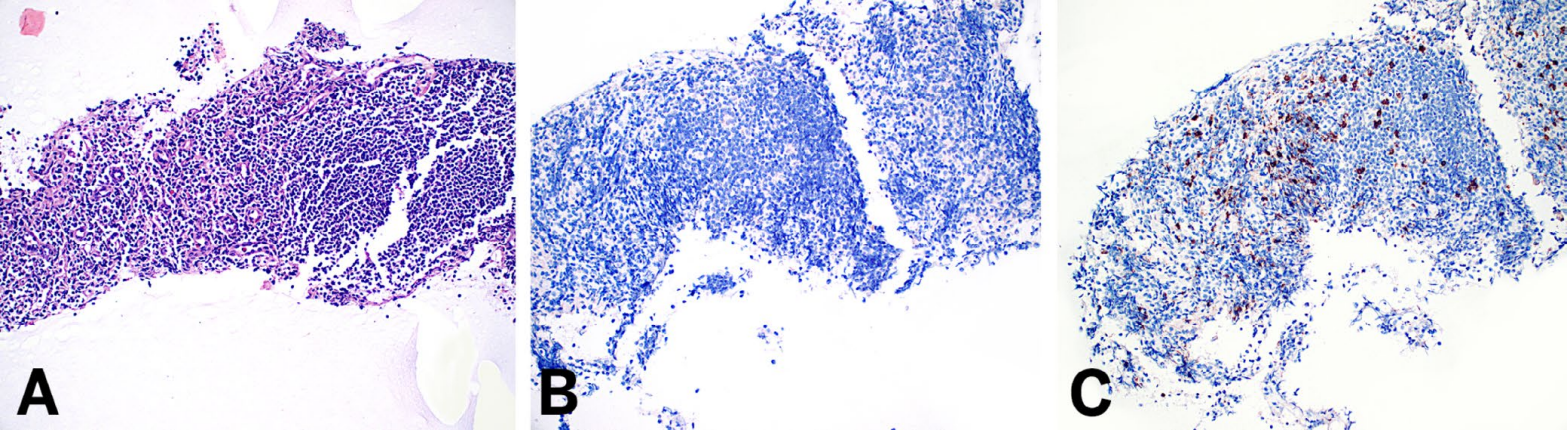
(A) H&E‐stained cell block showing an intra‐abdominal lymph node at 200× magnification. (B) SF1 immunostain is negative in the sample (200×). (C) Background T‐lymphocytes were positive for CD8 (200×). [Color figure can be viewed at wileyonlinelibrary.com]

The results of IHC staining for SF1 and CD8 across accessory spleen and lymph node cases are summarized in Table 2. In all 13 accessory spleen cases, SF1 demonstrated strong, uniform nuclear staining in a characteristic sinusoidal distribution (100%). This staining pattern was closely mirrored by CD8, which showed distinct cytoplasmic staining along the sinusoidal vasculature in all cases (100%) (Figure 2). In one case with limited cellularity, while SF1 and CD8 showed staining in a sinusoidal distribution, SF1 was easier to interpret due to its more distinct nuclear pattern (Figure 3). In the regional lymph node cases, neither SF1 nor CD8 demonstrated sinusoidal staining in any of the samples (0%) (Figure 4). CD8 highlighted scattered background T‐cells without the distinctive vascular pattern seen in splenic tissue.

**TABLE 2:** Immunohistochemical findings in accessory spleen cases (*N* = 13) versus regional lymph nodes (*N* = 15).

Marker	Staining pattern	Accessory spleen no. of positive cases, (%)	Regional lymph node no. of positive cases, (%)
SF1	Nuclear, sinusoidal	13 (100)	0 (0)
CD8	Cytoplasmic, sinusoidal	13 (100)	0 (0)

## 4 Discussion

The physiologic role of SF1 has been extensively studied in both human and animal models, particularly in the context of steroidogenesis within the adrenal glands and gonads. Studies in animal models have underscored the role of the SF1 gene in the development of splenic vasculature, showing that SF1 is expressed in specialized endothelial cells within the spleen [7]. However, this insight has not yet been translated to clinical practice for identifying accessory spleens in FNA samples. The diagnostic advantage of using SF1 immunostaining lies in its easily interpretable nuclear pattern, specificity for splenic endothelial LCs, and lack of reactivity in T cells and vascular structures in lymph nodes [8]. These highly specific features are particularly useful when dealing with limited cell block material.

Ectopic spleens are most commonly found near the splenic hilum, followed by the pancreatic tail [9]. Their recognition on FNA samples relies on being aware of their typical location and characteristic cytomorphologic features that include a preponderance of small, mature lymphocytes, a minor component of eosinophils and histiocytes, and vascular sinusoids [10]. IHC markers, such as CD8, which highlights the littoral sinusoidal endothelial cells, are typically used to support the diagnosis [10, 11]. Flow cytometry may be employed to exclude low‐grade lymphoproliferative processes that can affect splenules and hilar lymph nodes.

However, in FNA specimens with scant cellularity, the diagnostic utility of the morphologic and IHC features described above is limited. In this context, SF1 offers distinct advantages. Its highly specific expression in splenic sinusoids among lymphoid tissues, along with its strong nuclear staining pattern, is easier to interpret in small or poorly preserved specimens, enhancing diagnostic confidence. In our study, we found that both SF1 and CD8 demonstrated similar sensitivity in identifying ectopic splenic tissue. However, SF1 was significantly easier to interpret, particularly in limited or fragmented specimens.

One important diagnostic consideration is the potential for confusion with ectopic adrenal tissue. Ectopic adrenal tissue has been reported in many intra‐abdominal locations, including the celiac plexus, kidney, broad ligament, and liver [12]. While both adrenal and splenic tissues may express SF1, adrenal tissue has a distinct morphologic appearance that does not overlap with splenules. In cases with distorted morphology, additional markers such as Melan‐A, inhibin, and calretinin can help avoid diagnostic pitfalls.

On imaging, accessory spleens are most commonly mistaken for pancreatic neuroendocrine tumors (PanNETs) [4, 9, 13, 14]. Although PanNETs and splenic tissue are morphologically distinct, the possibility of misinterpretation remains, especially in cytology specimens where stripped neuroendocrine tumor cells may appear similar to lymphocytes. PanNETs can be confirmed with markers such as synaptophysin, chromogranin, and insulinoma‐associated protein 1 (INSM1). Peripancreatic lymph nodes constitute the other key differential based on cytomorphology. While lymph nodes closely mimic splenules, they lack the distinctive sinusoidal pattern of staining observed with the CD8 and SF1 immunostains.

Another theoretical advantage not formally tested in this study is the ease of interpreting nuclear stains on cell transfer compared to cytoplasmic stains. In the cell transfer technique, the coverslip is removed from an ethanol‐fixed, Papanicolaou‐stained slide, then a mounting medium is applied, and the slide is oven‐baked to embed the cells in the medium. After rehydration, the diagnostic material is transferred to a new slide where IHC can be conducted. Nuclear stains are preferred because they produce less background staining, which facilitates interpretation. This can be particularly helpful in hypocellular accessory spleen specimens or those lacking cell blocks.

In conclusion, the addition of SF1 to the diagnostic toolbox enhances the pathologist's ability to differentiate accessory spleens from potential mimics, especially in limited samples, thereby improving diagnostic accuracy and potentially preventing unnecessary surgical intervention or overtreatment.

## Author Contributions

**Omer Saeed:** conceptualization, methodology, data curation, formal analysis, writing – review and editing. **Adeyinka Akinsanya:** writing – original draft, writing – review and editing. **Jessica L. Muldoon:** data curation, writing – review and editing. **Mohamed Mustafa:** writing – review and editing. **Bin Yang:** writing – review and editing. **Hector Mesa:** writing – review and editing.

## Conflicts of Interest

The authors declare no conflicts of interest.

## Data Availability

The data that support the findings of this study are available on request from the corresponding author. The data are not publicly available due to privacy or ethical restrictions.
